# Clinical and Laboratory Characteristics of Pediatric COVID-19 Population—A Bibliometric Analysis

**DOI:** 10.3390/jcm11205987

**Published:** 2022-10-11

**Authors:** Ionela Maniu, George Maniu, Maria Totan

**Affiliations:** 1 Mathematics and Informatics Department, Research Center in Informatics and Information Technology, Faculty of Sciences, “Lucian Blaga” University, 5-7 Ion Ratiu Str., 550025 Sibiu, Romania; 2 Pediatric Research Department, Clinical Pediatric Hospital, 2-4 Pompeiu Onofreiu Str., 550166 Sibiu, Romania; 3 Faculty of Medicine, “Lucian Blaga” University of Sibiu, 2A Lucian Blaga Str., 550169 Sibiu, Romania; 4 Clinical Laboratory, Clinical Pediatric Hospital, 2-4 Pompeiu Onofreiu Str., 550166 Sibiu, Romania

**Keywords:** COVID-19, clinical, symptoms, laboratory, bibliometric, VOSviewer

## Abstract

The literature on the COVID-19 landscape has rapidly expanded in the pandemic period. The current study undertakes a bibliometric analysis of research in the topic of the clinical and laboratory characteristics of pediatric COVID-19 cases. Our aim is to perform a comprehensive bibliometric review of current research trends and patterns of this research domain. Publications retrieved from the Web of Science Core Collection and VOSviewer were used for analysis and network visualization. We analyzed geographical distribution and temporal trends, collaboration and citation patterns of authors, institutions, and countries, and core research themes from co-occurrence of keywords and terms. The analysis showed that contributions in the research field were from 302 publications, 1104 institutions, 62 countries, and 172 journals. Many publications were authored by American and Chinese authors, and many were published in the *Pediatric Infectious Disease Journal*, *Pediatric Pulmonology*, and *Frontiers in Pediatrics*. The top cited and co-cited journals were the *New England Journal of Medicine*, *Nature*, *JAMA*, *Lancet Infectious Diseases*, and *BMJ*. The network visualization maps of keywords and terms offered a global overview of the clinical and laboratory characteristics of pediatric COVID-19 patients. The bibliometric profile of the researched domain, based on analyzing a large collection of publications/data, could (i) enrich the researchers and non-researchers understanding of the field existing patterns and trends, and (ii) be useful in clinical practice (diagnostic and management) and public health policy.

## 1. Introduction

The literature on the COVID-19 landscape has rapidly expanded in the pandemic period.

Systematic reviews and meta-analysis studies are gathering existing knowledge, offering synthesized, selected, high-quality research evidence, seeking patterns and agreement or disagreement among studies results [1]. However, the productivity, impact, and trends can be pooled using bibliometric analysis.

The bibliometric analysis method systematically studies the specific topic (by conducting a statistical data analysis and content analysis) and determines its research growth, emerging topics discussed in different articles, and trends over time. By its indicators (quantity indicators measure the productivity of the researchers; quality indicators measure the performance of researchers output; structural indicators measure the connections between publications, authors, and areas of research), the bibliometric method provides quantitative and qualitative analysis and measurement of the specific research field [2].

Several reviews and meta-analysis summarized the clinical, laboratory, radiological features, outcome, and treatment of the SARS-CoV-2 infection at various time periods during the pandemic [3,4,5,6,7,8]. 

The aim of this study is to present a global overview of clinical and laboratory characteristics of pediatric COVID-19 cases using a bibliometric approach. We addressed the following research directions: to identify most frequently used keywords/terms (and their co-occurrence network) related to the research field, to identify the most productive countries and organizations, to identify sources where researchers publish their work, authorship, and collaborative patterns, and to identify influential research papers (highly cited). To the best of our knowledge, there is currently no bibliometric review related to the medical literature about the topic of clinical and laboratory characteristics of pediatric COVID-19 cases. 

## 2. Materials and Methods

**Figure 1:**
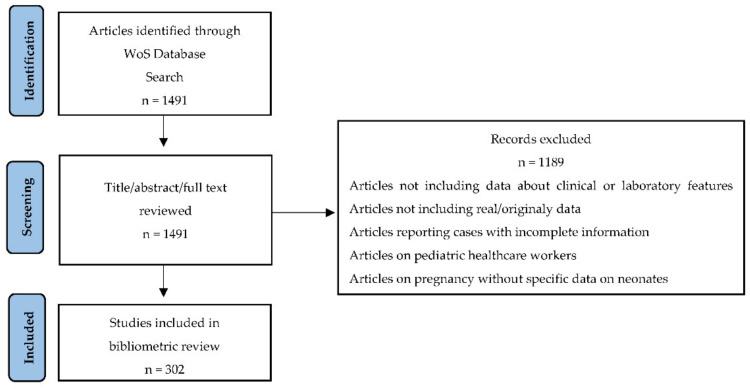
PRISMA flow chart of the study selection process.

We conducted a literature search on Web of Science Core Collection (WoS) online databases in October 2021 to identify scientific contributions, including analysis of clinical and/or laboratory characteristics of pediatric COVID-19 cases. The search identified publications that contain the terms (COVID-19 OR coronavirus OR SARS-CoV-2) and (pediatric OR children) and (laboratory or hematological or predict*) and (symptoms or clinical or signs or characteristic*) in their title or abstract or keywords or keywords plus. We included only article-type documents from 2020 to October 2021. MT and IM reviewed the titles, abstracts, and full texts of articles selected by the automated search to reach a consensus on inclusion of the topic-relevant ones. We included published papers that reported original data about the clinical or laboratory features of children with COVID-19. Included study designs were case-control, cohort studies, case reports (more than one case), and case series. Some studies included pediatric patients aged up to 18 years, while others included patients aged up to 20 years. We included studies considering both situations. The process of study selection was described using a PRISMA flow diagram, as in Figure 1.

Characteristics of each publication identified from the search include publication title, abstract, keyword, keyword plus, authorship, document type, publication year, journal title, language, journal category, and number of total citations. We used the VOSviewer science visualization software package developed by Van Eck and Waltman at the Center for Science and Technology Studies of Leiden University [9,10]. The bibliometric approach using this tool was applied in studies from various areas of research [11,12,13,14]. We performed the following types of bibliographic techniques: citation analysis, co-word analysis, and co-citation analysis. Networks were created based on co-relations between authors/institutions/countries/journals and co-occurrence of keywords or terms.

## 3. Results

Based on the literature search strategy, it was found that there are 302 publications, literature from the WoS database, comprising clinical or laboratory characteristics of pediatric COVID-19 cases.

**Figure 2:**
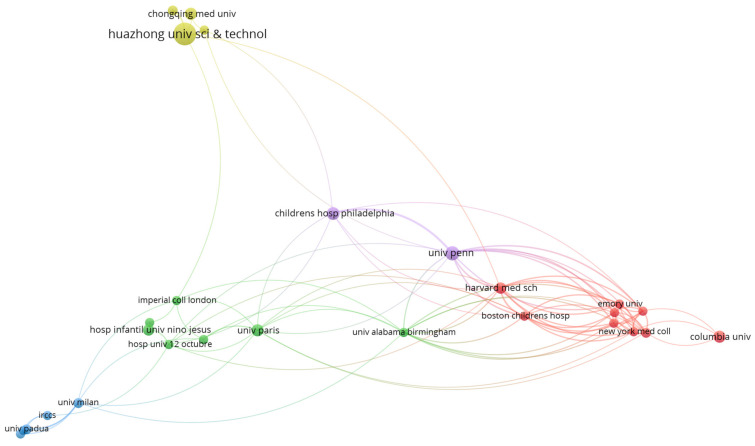
VOSviewer network visualization map of institutions/organizations (type of analysis: co-authorship, weights—documents, minimum number of documents of a country—5, 32 institutions meet the threshold, largest set of connected items—27). Groups of organizations that are strongly related to each other are represented using the same color (red, blue, green, etc.).

More than 1000 institutions (1104) from 62 countries/regions have reported clinical or laboratory characteristics of pediatric COVID-19 cases in their research. The most productive institutions were Huazhong Univ. of Science and Technology (29 studies, 1359 citations, TLS 687), the Univ. of Pennsylvania (11 studies, 1573 citations, TLS 751), the Children’s Hospital of Philadelphia (9 studies, 816 citations, TLS 280), Univ. Paris (8 studies, 616 citations, TLS 536), Columbia Univ. (8 studies, 170 citations, TLS 226), and Chongqing Med. Univ. (8 studies, 66 citations, TLS 134). The most cited institutions were the Univ. of Pennsylvania (1573 citations, 11 studies, TLS 751), Huazhong Univ. of Science and Technology (1359 citations, 29 studies, TLS 687), Vanderbilt Univ. (896 citations, 5 studies, TLS 635), Harvard Med. School (874 citations, 7 studies, TLS 706), and Boston Children’s Hospital (862 citations, 5 studies, TLS 693). Collaboration analysis of the institutions is presented in Figure 2, with wider lines indicating stronger collaboration.

**Figure 3:**
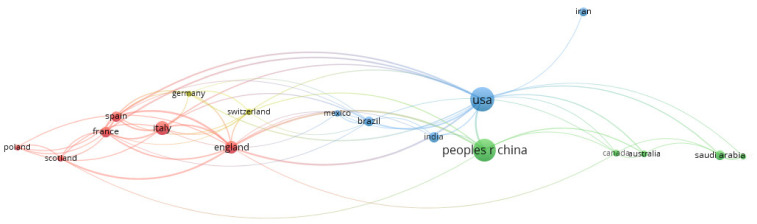
VOSviewer network visualization map of countries (type of analysis: co-authorship, weights—documents, minimum number of documents of a country—5, 19 countries meet the threshold, largest set of connected items—18 countries—Turkey meets the threshold (5 documents) but is not connected with other countries).

The leading countries in the analyzed researched were the USA (76 studies, 3751 citations), China (67 studies, 3426 citations), Turkey (41 studies, 90 citations), Italy (26 studies, 493 citations), England (21 studies, 946 citations), and France (14 studies, 727 citations). The rank of the countries is detailed in Table 1, while Figure 3 highlights the country/region co-authorship network.

**Figure 4:**
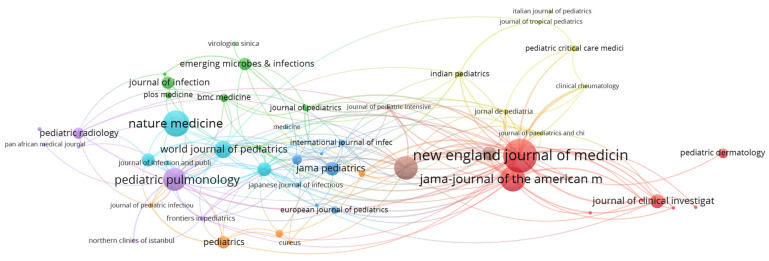
VOSviewer network visualization map of sources (type of analysis: citation, weights—citations, minimum number of documents of a source—2, 55 sources meet the threshold, largest set of connected items—53 sources).

Top productive and cited journals that include publication related to clinical and laboratory characteristics of pediatric COVID-19 cases are presented in Table 2 and Figure 4. These journals focus on research areas such as general and internal medicine, biochemistry and molecular biology, cell biology, research and experimental medicine, infectious diseases, pediatrics, immunology, the respiratory system, and virology. 

The top 20 highly cited articles are presented in Table 3. To observe the evolution/trend over time of the number of citations, a hierarchy of the most-cited articles observed on 8 October 2021 was presented, as well as the hierarchy of citations for a period of time approximately one year later (in 20 June 2022). Multisystem inflammatory syndrome in children was a topic of paramount interest. 

**Figure 5:**
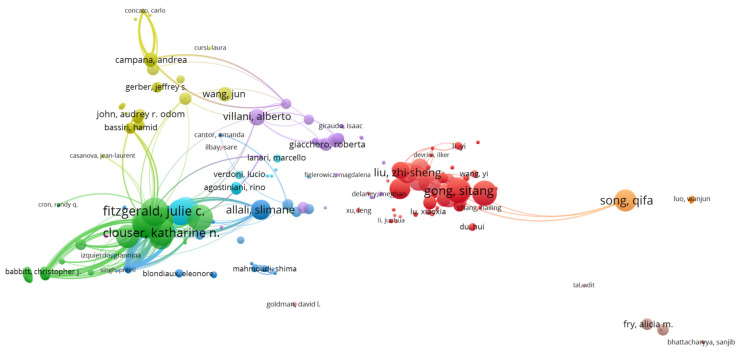
VOSviewer network visualization map of authors (type of analysis: citation, weights—citations, minimum number of documents of an author—2399 authors meet the threshold, largest set of connected items—396 sources).

Figure 5 presents the network visualization map of authors. Among 3509 authors, the most productive were:

- Shao, Jianbo (Wuhan Maternal and Child Healthcare Hospital, Tongji Medical College, Huazhong University of Science and Technology, China, 8 documents, 588 citations, 66 TLS)
- Li, Hui (Department of Hematology, Wuhan Maternal and Child Healthcare Hospital, Tongji Medical College, Huazhong University of Science and Technology, 7 documents, 368 citations, 72 TLS)
- Villani, Alberto (Department of Emergency, Acceptance and General Pediatrics, Bambino Gesù Children’s Hospital, Rome, Italy, 6 documents, 299 citations, 115 TLS)
- Rossi, Paolo (Academic Department of Pediatrics, Research Unit of Clinical Immunology and Vaccinology, Bambino Gesù Children’s Hospital, Chair of Pediatrics, Department of Systems Medicine, University of Rome “Tor Vergata,” Rome, Italy, 5 documents, 181 citations, 110 TLS)
- Xiao, Han (Institute of Maternal and Child Health, Wuhan Children’s Hospital, Tongji Medical College, Huazhong University of Science and Technology, Wuhan, China, 5 documents, 337 citations, 45 TLS)

The authors with the highest number of citations were:

- Fitzgerald, Julie (Department of Anesthesiology and Critical Care Medicine, The Children’s Hospital of Philadelphia, Philadelphia, USA, 4 documents, 862 citations, 176 TLS)
- Son, Mary Beth F. (Division of Immunology, Department of Pediatrics, Boston Children’s Hospital, Boston, USA, 4 documents, 858 citations, 168 TLS)
- Newburger, Jane (Department of Cardiology, Boston Children’s Hospital, Department of Pediatrics, Harvard Medical School, Boston, USA, 3 documents, 839 citations, 121 TLS)
- Clouser, Katharine (Hackensack University Medical Center, New Jersey, USA, 4 documents, 788 citations, 173 TLS)

**Figure 6:**
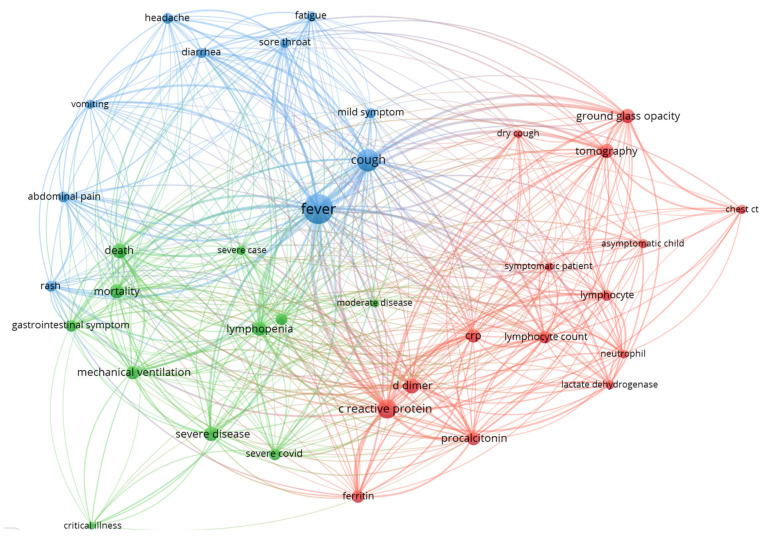
VOSviewer network visualization map of keywords (author keywords and keywords plus)—symptoms and laboratory related keywords (encountered minimum 10 times). A more detailed figure is Supplementary Figure S1 where, from the total number of 645 keywords, 160 were encountered a minimum 2 times.

**Figure 7:**
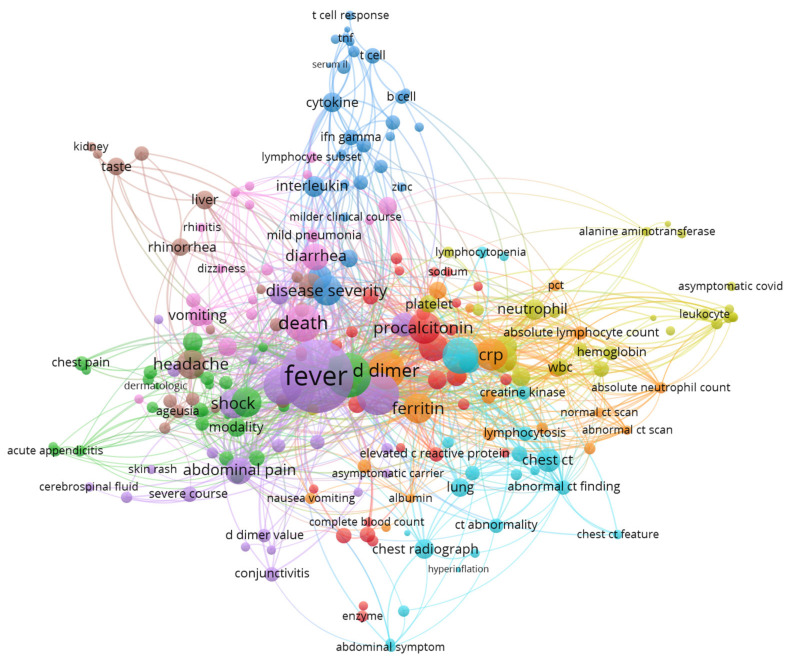
VOSviewer network visualization map of terms, from title and abstract—symptoms and laboratory related keywords (encountered a minimum of 2 times). A more detailed figure is Supplementary Figure S2 where, from the total number of 6466 terms, 242 were encountered a minimum of 10 times.

Keywords networks are usually used to map topics of the research field. The knowledge map of keywords co-occurrence (author keywords and keywords plus) and knowledge map of terms from publication title and abstract are presented in Figure 6 and Figure 7 and Figures S1 and S2. The visualizations show the covered topics related to symptoms, laboratory, and imaging features of pediatric COVID-19 cases and are summarized in Table 4.

## 4. Discussion

This study reveals the bibliometric profile of the research and visualization of connections between pieces of information in the field of clinical and laboratory characteristics of pediatric population with COVID-19. It summarizes the most frequent symptoms and laboratory parameters of the literature in the research field. The literature in the studied field is represented by more than 300 publications. On the list of top productive countries were the USA, China, Turkey, Italy, and England, while on the list of top cited countries were the USA, China, England, France, and Italy. 

Keyword/term-based maps show a global overview of clinical and laboratory characteristics of pediatric COVID-19 cases. A network visualization map of terms and keywords revealed the most commonly included symptoms and laboratory imaging findings among pediatric patients with COVID-19. Regarding symptoms, terms related to systemic, respiratory, gastrointestinal, neurological, olfactory, dermatological, ocular system, rheumatic, and cardiac symptoms were observed. Regarding laboratory features, terms related to inflammatory markers, immune markers, d-dimer levels, and liver enzymes were observed.

Terms like multisystem inflammatory syndrome (52), MISC/MIS-C (48/17), Kawasaki disease/Kawasaki-disease/Kawasaki-like disease (11/10/4), macrophage activation syndrome/MAS (7/4), and toxic shock syndrome/TSS (8/2) are referring to studies that describe clinical or laboratory values of coronavirus disease 2019-related multisystem inflammatory syndrome in children or Kawasaki disease or TSS or MAS. Among MISC-related studies, some of them were addressing organ damage or impairment (liver [35], cardiac [32,36,37,38,39], and neurologic [40]).

Some studies described/compared clinical or laboratory values among asymptomatic, mild, moderate, severe cases [41,42,43,44,45], survivors and non-survivors [46,47], different age groups [43], and children and adults [48,49]. Some studies described clinical or laboratory characteristics of neonates and/or infants [39,50,51].

Relevant organizations in this field of study, in terms of production and citation, were from China—Huazhong University of Science and Technology from Wuhan, Whan University, Chongqing Medical University; the USA—Children’s Hospital of Philadelphia, University of Pennsylvania, Columbia University; France—Université de Paris, Necker-Enfants Malades University Hospital; Spain—Hospital Infantil Universitario Niño Jesús; Turkey—University of Health Science; and Italy—University of Milan.

The studies of researchers from the two institutions in Wuhan covered several directions. The clinical and laboratory characteristics of patients were analyzed in case of neonates [51] and neonates born from mothers with or without COVID-19 [52], patients with different severities and allergic statuses [53], prolonged duration of viral shedding [41], family clusters [25,54], and inflammatory responses to COVID-19 infection [55,56]. Most of these studies referred to patients from the Wuhan Children’s Hospital, but there were also studies that referred to patients from other hospitals [25,57]. 

Studies published in 2021, compared to studies published in 2020, included differentiation/comparisons of the clinical and laboratory features of children between COVID-19 and non-COVID-19 pediatric pneumonia [58], and COVID-19 and the influenza virus [59], as well as multicenter studies [23,58,60]. Studies published at the beginning of the pandemic, immediately after the appearance of the first cases [18,19], presented routine clinical and laboratory characteristics. With the evolution of the pandemic, more parameters were analyzed (serum cytokines IL-2, IL-4, IL- 6, IL-10, TNF-α, IFN-γ; lymphocyte subsets: T cells, CD3+T cell, CD4+T cell, CD3+CD4+T cell, CD3+CD8+T cell, CD4+/CD8+T ratio, NK (natural killer), CD4+CD25+T cell, B cell, and CD19+B cell [53,56,61]). In the study [56], the results showed that the levels of pro-inflammatory cytokines (IL-2, IL-4, IL-6, TNF-α, and IFN-γ) were not different between mild, moderate, and severe/critical groups of children with COVID-19. In addition, the numbers of CD4+T cells, CD8+T cells, B cells, and NK cells are not reduced, even in moderate or severe disease. In the study [59,62], COVID-19 pediatric patients had lower severity of illness (clinical profile and outcomes) than children with influenza: lymphocyte count and D-dimer were lower in influenza A patients than in COVID-19, and the severity index of pneumonia (CRP, PCT (procalcitonin)) was lower in COVID-19 than influenza A. Ground-glass opacity was more common in COVID-19 patients than in influenza A patients, and consolidation was more frequent in influenza A patients [62]. The study [63], reporting results of 1-month follow-up of clinical features, highlighted the association between involvement of different lung lobes and persistence of pneumonia, elevation of creatinine kinase and CK-MB isoenzyme levels, and body temperature. The study [16] suggests that the gastrointestinal tract may shed the virus and fecal–oral transmission may be possible (persistently positive real-time RT–PCR tests of rectal swabs after their nasopharyngeal testing had become negative). 

In the USA, the studies of researchers from the Children’s Hospital of Philadelphia analyzed MISC and/or Kawasaki disease [64,65], acral pernio-like skin rashes concurrent with COVID-19 [66], convalescent plasma for COVID-19 pediatric patients [67], and the epidemiology of COVID-19 in a multicenter study [68]. The research direction at Columbia University included MISC and/or COVID-19 diseases and liver involvement—elevated ALT [69], and cardiac abnormalities [39]. The study [64], which analyzed the differentiation between minimal COVID-19, severe COVID-19, and MIS-C, showed that the combination of the cytokine levels (IL-10 and TNF-α), together with the presence of burr cells and toxic granulation on peripheral blood smears, discriminate between MIS-C and severe COVID-19 cases. In the study [15], with 186 MISC patients from 26 states of the United States, most children (92%) had elevations in at least four inflammation biomarkers while organ-system involvement included the gastrointestinal (92%), cardiovascular (80%), hematologic (76%), mucocutaneous (74%), and respiratory system (70%). In the study [66] on acral changes (pernio-like skin rashes—feet, hand, and head/neck lesions) concurrent with COVID-19, all patients recovered (lesions lasted 3 weeks, on average) and had no short-term sequelae. Only a few children had blood-test abnormalities and, in 41% of cases, positive antinuclear antibody (ANA) titers were found. The authors suggest that this phenomenon is not only due to temporal conditions (spring months/cold environments) or virus wave but can be a late-phase reaction of the infection (skin changes appeared, on average, after 2–3 weeks of initial COVID-19 symptoms or positive test). 

Research by authors affiliated to Hospital Infantil Universitario Niño Jesús from Spain investigated gastrointestinal (GI) manifestations [70,71], cardiovascular manifestations [36], and skin manifestations (chilblains) [72] in COVID-19 patients (including MISC disease). In an observational study conducted in 15 hospitals in Spain [70], results showed that patients with digestive symptoms tended to have higher CRP and PCT. An increased percentage of MISC patients showed gastrointestinal symptoms. Frequency of GI has increased with the progression of the pandemic, not only because of the growing awareness of clinicians but also because of the virus mutations. Digestive symptoms usually appear before the onset of fever or respiratory symptoms (even in absence of respiratory signs) early during the course of COVID-19 and may lead to a diagnostic delay (gastroenteritis of any etiology).

In Italy, findings of an multicentric study (11 pediatric hospitals predominantly from central and northern regions) [31] showed a favorable clinical course of COVID-19 infection in pediatric patients: the most common symptoms were fever and GI manifestations; there were rare cases of leukopenia, neutropenia, lymphopenia, and increased CK or LDH values. Reference [73] analyzed the plasmatic levels of inflammatory chemokines and cytokines in patients with COVID-19 and MIS-C and encountered higher levels of IL-6, CXCL8, CCL2/MCP-1, CXCL9/MIG, and CXCL10/IP-10 in MIS-C and higher levels of CCL5 in COVID-19, concluding that immunological events together with neutrophil activation might induce the multisystem and cardiovascular damage in MIS-C disease. In a study [24] with children from Italy and Sweden (presenting COVID, MIS-C, Kawasaki disease, and healthy controls), MIS-C patients had more pronounced lymphopenia, higher levels of CRP and ferritin, and lower platelet counts compared to children with SARS-CoV-2 infection or Kawasaki disease.

Our study had several limitations. We only included studies extracted and analyzed from the WoS database. Citations of research papers accumulate over time, being considered to be biased toward old articles (articles published recently are hardly cited (yet)). Although keyword and terms analysis offered relevant information regarding each research study, a more in-depth content analysis of the publications could provide more specific information/associations/mechanisms/etc. regarding COVID-19 pediatric features. Our study included only articles published until October 2021, though research on COVID-19 is still ongoing. In addition, we did not consider/ensure the methodological quality of the studies included in the bibliometric review.

We believe this analysis provides a global scientific output of clinical and laboratory characteristics that could be useful in informed research, clinical practice (diagnostic and management), and public health policy.

## Figures and Tables

**Table 1:** Statistics by countries.

Rank	Documents	Citations	Total Link Strength		
			Co-Authorship	Citations	Bibliographic Coupling
1	USA (76)	USA (3751)	USA (73)	USA (434)	USA (28,680)
2	China (67)	China (3426)	England (65)	China (251)	China (19,916)
3	Turkey (41)	England (946)	Italy (46)	England (207)	Turkey (17,967)
4	Italy (26)	France (727)	Spain (45)	Turkey (119)	Italy (11,416)
5	England (21)	Italy (493)	France (36)	France (107)	England (10,435)
6	India; Spain (15)	Scotland (322)	Brazil (33)	Italy (92)	France (7761)
7	France (14)	Spain (230)	Germany; China (26)	Scotland (79)	Brazil (7381)
8	Brazil; Saudi Arabia (12)	Sweden (184)	Scotland (24)	Brazil (54)	India (6718)
9	Iran (11)	Wales (152)	Argentina; Peru; Switzerland (23)	Spain (48)	Spain (6627)
10	Scotland (6)	Turkey (90)	Chile (22)	India (41)	Saudi Arabia (5465)

**Table 2:** Top productive and cited journals, most co-cited sources.

Rank	Most Productive Journals Documents/Citations/ TLS/IF2020; WoS Category, JCR	Most Cited Journals Citations/Documents/ TLS/IF2020; WoS Category, JCR	Most Co-Cited Journals Citations/TLS/ IF2020; WoS Category, JCR
1	Pediatric Infectious Disease Journal 14/189/45/2.129 immunology, infectious disease, pediatrics Q3	New England Journal of Medicine 1070/2/86/91.245 medicine, general & internal Q1	New England Journal of Medicine 481/8705/91.245 medicine, general & internal Q1
2	Pediatric Pulmonology 11/492/50/3.039 pediatrics Q2, respiratory system Q3	Nature Medicine 665/2/11/53.440 biochemistry & molecular biology, cell biology, medicine, research & experimental Q1	Lancet Infectious Disease 323/5861/25.071 infectious diseases Q1
3	Frontiers in Pediatrics 10/26/18/3.418 pediatrics Q1	Jama—Journal of American Medical Association 604/2/48/56.272 medicine, general & internal Q1	Pediatrics 299/5458/7.124 pediatrics Q1
4	Journal of Medical Virology 7/173/15/2.327 virology Q3	Lancet Infectious Disease 538/1/52/25.071 infectious diseases Q1	JAMA—Journal of American Medical Association 287/5330/56.272 medicine, general & internal Q1
5	World Journal of Pediatrics 6/298/32/2.764 pediatrics Q2	BMJ—British Medical Journal 526/2/38/39.890 medicine, general & internal Q1	JAMA Pediatrics 143/2837/16.193 pediatrics Q1

IF—impact factor, Q—quartile in category, JCR—journal citations reports, TLS—total link strength.

**Table 3:** Top 20 highly cited articles.

First Author Year [ref.]	Document Title	Journal	Citations (Rank) 8 October 2021/ 20 June 2022	Links
Feldstein 2020 [15]	Multisystem Inflammatory Syndrome in US Children and Adolescents	NEW ENGLAND JOURNAL OF MEDICINE	695 (1)/ 1066 (1)	46
Xu 2020 [16]	Characteristics of pediatric SARS-CoV-2 infection and potential evidence for persistent fecal viral shedding	NATURE MEDICINE	658 (2)/ 789 (3)	11
Whittaker 2020 [17]	Clinical Characteristics of 58 Children with a Pediatric Inflammatory Multisystem Syndrome Temporally Associated With SARS-CoV-2	JAMA—JOURNAL OF THE AMERICAN MEDICAL ASSOCIATION	554 (3)/ 851 (2)	41
Qiu 2020 [18]	Clinical and epidemiological features of 36 children with coronavirus disease 2019 (COVID-19) in Zhejiang, China: an observational cohort study	LANCET INFECTIOUS DISEASES	538 (4)/ 645 (4)	52
Xia 2020 [19]	Clinical and CT features in pediatric patients with COVID-19 infection: Different points from adults	PEDIATRIC PULMONOLOGY	462 (5)/ 550 (6)	36
Toubiana 2020 [20]	Kawasaki-like multisystem inflammatory syndrome in children during the COVID-19 pandemic in Paris, France: prospective observational study	BMJ	395 (6)/ 508 (7)	24
Dufort 2020 [21]	Multisystem Inflammatory Syndrome in Children in New York State	NEW ENGLAND JOURNAL OF MEDICINE	375 (7)/ 579 (5)	42
Sun 2020 [22]	Clinical features of severe pediatric patients with coronavirus disease 2019 in Wuhan: a single center’s observational study	WORLD JOURNAL OF PEDIATRICS	277 (8)/ 336 (8)	23
Zheng 2020 [23]	Clinical Characteristics of Children with Coronavirus Disease 2019 in Hubei, China	CURRENT MEDICAL SCIENCE	190 (9)/ 227 (13)	9
Consiglio 2020 [24]	The Immunology of Multisystem Inflammatory Syndrome in Children with COVID-19	CELL	174 (10)/ 304 (9)	14
Su 2020 [25]	The different clinical characteristics of corona virus disease cases between children and their families in China—the character of children with COVID-19	EMERGING MICROBES & INFECTIONS	153 (11)/ 177 (16)	10
Liu 2020 [26]	Clinical and CT imaging features of the COVID-19 pneumonia: Focus on pregnant women and children	JOURNAL OF INFECTION	137 (12)/ 196 (15)	3
Kim 2020 [27]	Hospitalization Rates and Characteristics of Children Aged < 18 Years Hospitalized with Laboratory-Confirmed COVID-19-COVID-NET, 14 States, 1 March–25 July 2020	MMWR—MORBIDITY AND MORTALITY WEEKLY REPORT	132 (13)/ 241 (12)	12
Swann 2020 [28]	Clinical characteristics of children and young people admitted to hospital with COVID-19 in United Kingdom: prospective multicentre observational cohort study	BMJ-BRITISH MEDICAL JOURNAL	131 (14)/ 253 (10)	16
Davies 2020 [29]	Intensive care admissions of children with paediatric inflammatory multisystem syndrome temporally associated with SARS-CoV-2 (PIMS-TS) in the UK: a multicentre observational study	LANCET CHILD & ADOLESCENT HEALTH	129 (15)/ 203 (14)	11
Li 2020 [30]	Chest computed tomography in children with COVID-19 respiratory infection	PEDIATRIC RADIOLOGY	120 (16)/ 121 (20)	12
Garazzino 2020 [31]	Multicentre Italian study of SARS-CoV-2 infection in children and adolescents, preliminary data as at 10 April 2020	EUROSURVEILLANCE	116 (17)/ 148 (18)	16
Grimaud 2020 [32]	Acute myocarditis and multisystem inflammatory emerging disease following SARS-CoV-2 infection in critically ill children	ANNALS OF INTENSIVE CARE	109 (18)/ 155 (17)	6
Zachariah 2020 [33]	Epidemiology, Clinical Features, and Disease Severity in Patients with Coronavirus Disease 2019 (COVID-19) in a Children’s Hospital in New York City, New York	JAMA PEDIATRICS	107 (19)/ 248 (11)	23
Zhang 2020 [34]	Detectable SARS-CoV-2 viral RNA in feces of three children during recovery period of COVID-19 pneumonia	JOURNAL OF MEDICAL VIROLOGY	106 (20)/ 119 (19)	5

**Table 4:** Terms/keywords related to the topic of clinical and laboratory characteristics of pediatric COVID-19 cases.

	Terms Related to:
**Symptoms**	
Systemic	Fever (133), fatigue (14), myalgia (8), anemia (4)
Respiratory	Cough (75), sore throat (17), dry cough (10), dyspnea (9), tachypnea (6), rhinorrhea (8), runny nose (4), nasal congestion (4), rhinitis (3), nasal discharge (2)
Gastrointestinal	Gastrointestinal (32), diarrhea/diarrhea (21/4), abdominal pain/discomfort/symptoms (18/2/2), vomiting (14), nausea (3)
Neurological	Headache (22), dizziness (2), irritability (2), encephalopathy (4), hypoxia (2)
Olfactory	Taste (8), smell/anosmia (6/3), ageusia/dysgeusia (4/3)
Dermatological	Rash (19), dermatologic (2), skin/mucocutaneous rash (3/2), cheilitis (2)
Ocular	Conjunctivitis (5), conjunctival injection (5)
Rheumatic	Juvenile idiopathic arthritis (5), arthritis rheumatol (15)
Cardiac	Myocarditis (10), hypertension (8), chest pain (5), cardiac arrhythmia (3), tachycardia (2)
**Laboratory markers**	
Biochemistry	C reactive protein/CRP (72/26), lactate dehydrogenase/LDH (15/12), ALT/alanine aminotransferase (7/2), aspartate aminotransferase/AST (3/4), creatinine kinase/creatine kinase mb (4/3), sodium/hyponatremia (2/2), zinc (3), total bilirubin (3), albumin/hypoalbuminemia (2/2), alpha hydroxybutyrate dehydrogenase (2)
Cardiac	B-type natriuretic peptide/BNP (6/3), troponin (4)
Coagulation	D-dimer (43), fibrinogen (10), prothrombin time (2)
Hematology	Lymphocyte/lymphopenia/lymphocytosis/lymphopenium/lymphopaenia (44/36/5/3/2), absolute lymphocyte count (4), procalcitonin (30), neutrophil/neutropenia (19/5), absolute lymphocyte count (3), platelet (15), WBC/white blood cell count (8/7), thrombocytopenia (10), T cell/T cell response (6/2), CD4 (5), CD8 T cell (3), B cell (4), MPV (8), erythrocyte sedimentation rate/ESR (5/4), hemoglobin (5), leukocytosis/leukopenia/leukocyte (4/4/3), neutrophil lymphocyte ratio (3), hematologic (3)
Immunology	Ferritin (27), interleukin/IL-6/interleukin-6 (13/4/4), chemokine (4), IFN gama/interferon gamma (10/3), cytokine storm (10), TNF alpha (9), Th Ts (2), immunoglobulin/IgG/IgM/IgA (12/7/7/3), total immunoglobulin E (2)
Treatment	Mechanical ventilation (19), invasive/noninvasive ventilation (8/3), oxygen therapy (4), antiviral therapy (3), azithromycin (3), corticosteroid (12), hydroxychloroquine (2), interferon alfa (1), intravenous immunoglobulin (12), lopinavir ritonavir (3), methylprednisolone (2), ribavirin (2), ritonavir (2), glucocorticoid (4), anakinra (5), antibiotic (4)
Coinfections	Influenza A (4), respiratory syncytial virus (4), influenza virus (2), mycoplasma pneumonia (2), adenovirus (2), rhinovirus (3)
Radiological/imaging	Chest CT finding/scan/examination/image/feature (6/5/4/3/2), chest radiograph/radiography/x-ray/imaging/finding/feature (10/5/9/8/6/4), abnormal CT finding/scan (5/2), abnormal radiological finding (2), lung (9), lung injury/involvement/lobe (6/4/3), ground glass opacity (29), patchy shadow (4), bilateral involvement/bilateral pneumonia (3/2), pleural effusion (3), interstitial opacity (2), sub-pleural area (2).

## Data Availability

The data are available on request from the corresponding author.

## Supplementary Materials

The following supporting information can be downloaded at: https://www.mdpi.com/article/10.3390/jcm11205987/s1, Figure S1: VOSviewer network visualization map of keywords (author keywords and keywords plus). From the total number of 645 keywords, 160 were encountered minimum 2 times; Figure S2: VOSviewer network visualization map of terms from title and abstract. From the total number of 6466 terms, 242 were encountered minimum 10 times.
