# Supplementary material for: Clinical and Laboratory Characteristics of Pediatric COVID-19 Population—A Bibliometric Analysis
Source: J Clin Med. 2022 Oct 11;11(20):5987. doi: 10.3390/jcm11205987 (PMC9605229; doi:10.3390/jcm11205987)
Supplement: Supplementary file 1 [file jcm-11-05987-s001.zip › jcm-1870818-supplementary.pdf]

Supplementary Materials

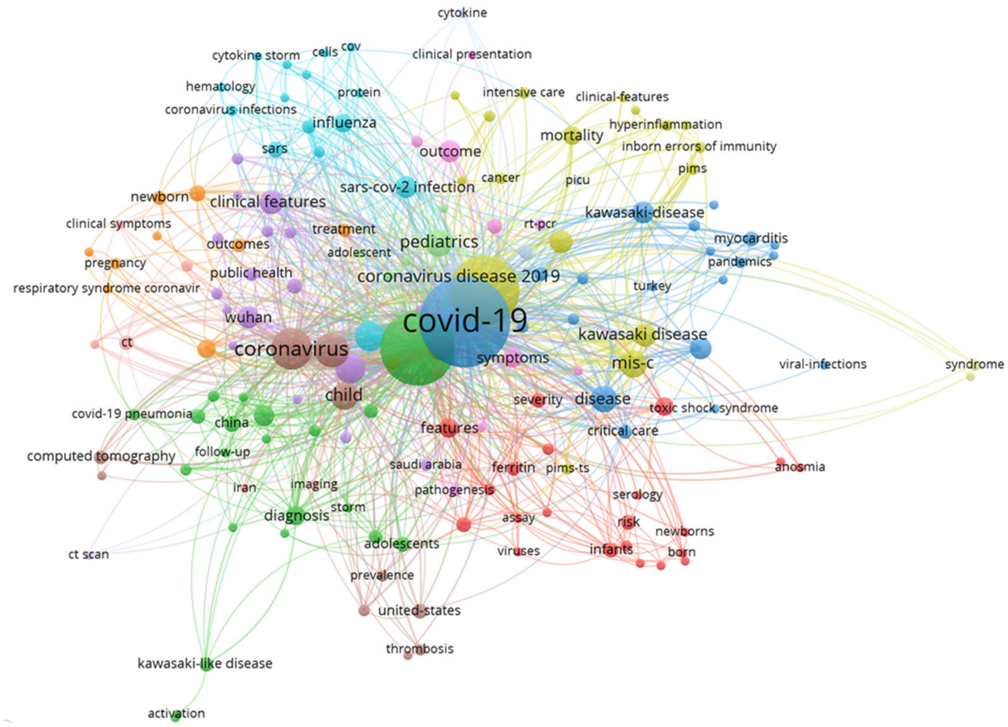

Figure S1. VOSviewer network visualization map of keywords (author keywords and keywords plus). From the total number of 645 keywords, 160 were encountered minimum 2 times.

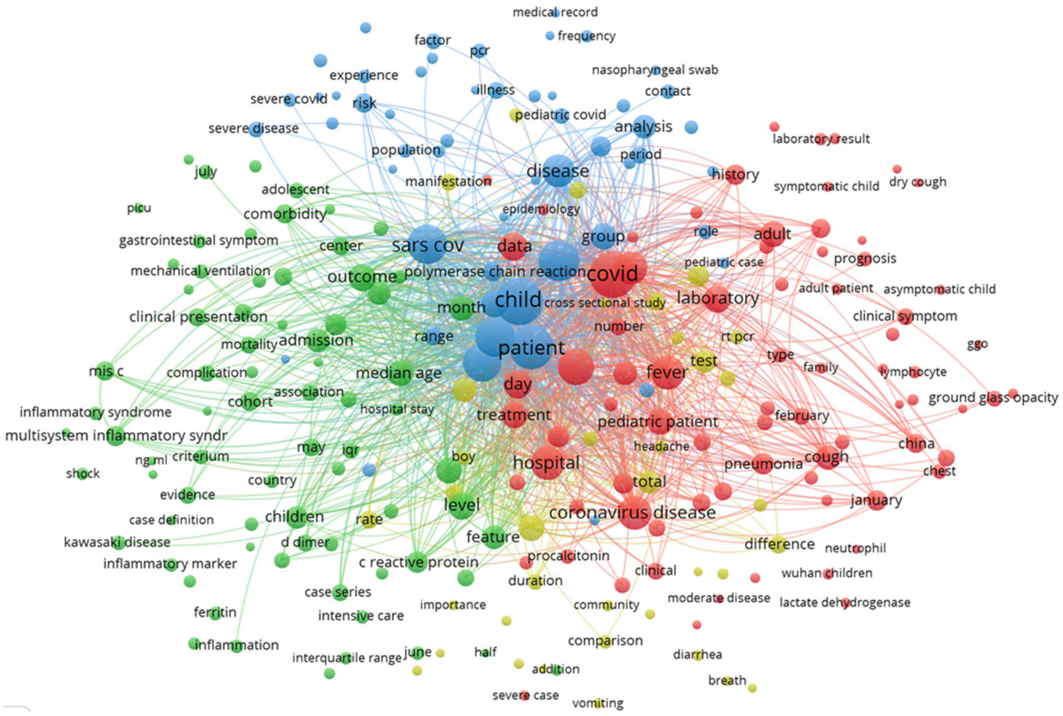

Figure S2. VOSviewer network visualization map of terms from title and abstract. From the total number of 6466 terms, 242 were encountered minimum 10 times.
